# Non-metal single atoms anchored on defective MoS_2_: a novel electrocatalyst for NO reduction to NH_3_

**DOI:** 10.1039/d5ra04718h

**Published:** 2025-08-19

**Authors:** Yifan Liu, Mamutjan Tursun, Guangzhi Hu, Abdukader Abdukayum, Chao Wu

**Affiliations:** a Xinjiang Key Laboratory of Novel Functional Materials Chemistry, College of Chemistry and Environmental Sciences, Kashi University Kashi 844000 PR China mmtj15@stu.xjtu.edu.cn; b Qilu Lake Field Scientific Observation and Research Station for Plateau Shallow Lake in Yunnan Province, Institute for Ecological Research and Pollution Control of Plateau Lakes, School of Ecology and Environmental Science, Yunnan University Kunming 650504 China; c Frontier Institute of Science and Technology, Xi'an Jiaotong University Xi'an 710054 PR China chaowu@xjtu.edu.cn

## Abstract

The electrocatalytic nitric oxide reduction reaction (eNORR) is a highly significant because it provides a sustainable and cost-effective way to combine the elimination of nitric oxide (NO) with synthesis of ammonia (NH_3_). This study comprehensively investigates the performance of single non-metal atom catalysts (NM@MoS_2_), which are composed of single non-metal atoms that are embedded in vacancy defects in MoS_2_. Our results demonstrate that eight NM@MoS_2_ catalysts (NM = B, C, N, O, P, Si, Se, and Te) exhibit remarkable thermodynamic stability. The Si, C, N, B and P@MoS_2_ catalysts in particular effectively adsorb and activate NO molecules, displaying high catalytic activity during the subsequent protonation process. Their *U*_L_ values are 0, 0, −0.36, −0.62, and −0.70 V, respectively. Furthermore, a detailed selectivity analysis revealed that the N, P, C, and Si@MoS_2_ catalysts exhibit high NH_3_ selectivity. This theoretical study has effectively identified and evaluated NM@MoS_2_ catalysts based on stability, selectivity and high catalytic activity with a focus on NO removal and NH_3_ synthesis.

## Abstract 2

The eNORR performance of the MoS_2_ catalyst doped with non-metal atoms has been studied. The Si, C, N, B, and P@MoS_2_ catalysts demonstrated great potential for eNORR. The Si, C, and B@MoS_2_ catalysts inhibited the formation of byproducts and HER.

## Graphical abstract

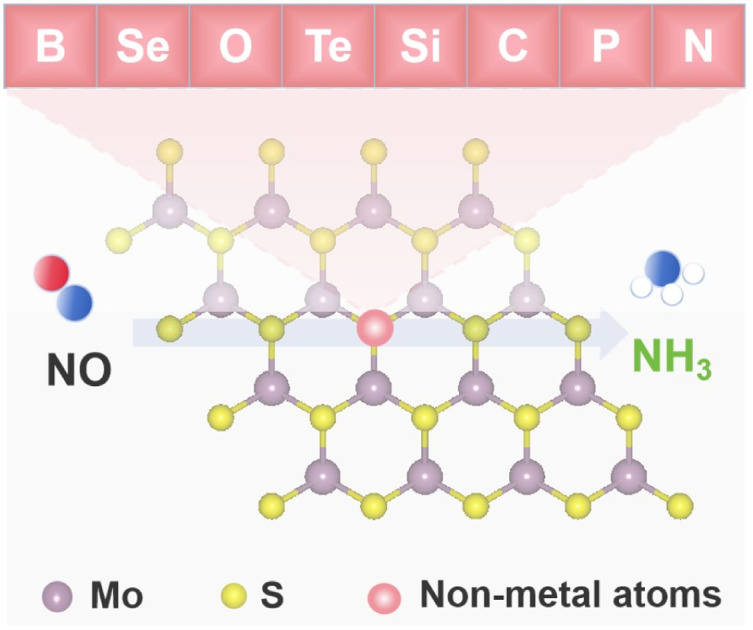

## 1 Introduction

Fossil fuel combustion in power plants, cars and industrial facilities releases large amounts of nitrogen oxides (NO_*x*_), which endanger the environment and human health.^1,2^ As public awareness of the pollution and health risks associated with NO_*x*_ emissions increases, more stringent regulations are being enacted across various industries to limit NO_*x*_ emissions.^3^ Consequently, eliminating NO_*x*_ from flue gases has become a key area of research within catalytic chemistry.

Currently, selective catalytic reduction (SCR) technology is the primary method for reducing NO_*x*_ discharges.^4–9^ However, this approach is hindered by its high energy consumption, which makes it unsuitable for widespread application. For example, the SCR process usually uses ammonia as a reducing agent and operates at temperatures between 200 and 400 °C. Meanwhile, ammonia (NH_3_), a vital chemical for fertilizers, pharmaceuticals, and dyes, is predominantly synthesised *via* the Haber–Bosch method, which demands intensely high temperatures (300–500 °C) and pressures (200–300 atm).^10^ These harsh conditions contribute to high energy costs and limit the sustainability and scalability of NH_3_ production.

Nitric oxide (NO) typically accounts for around 95% of total nitrogen oxides (NO_*x*_) in flue gas emissions.^11^ Recently, the electrocatalytic nitric oxide reduction reaction (eNORR) has emerged as an innovative, environmentally friendly solution for reducing NO emissions from sources such as thermal power stations, industrial facilities and vehicles. This process is advantageous because it operates at ambient temperatures, eliminating the need for high temperatures or pressures.

In addition, the eNORR process can utilize renewable energy sources like solar or wind power, boosting its environmental credentials. Another benefit of the eNORR process is its ability to produce valuable NH_3_.^12^ Therefore, eNORR is a win–win strategy for synthesising valuable NH_3_ and reducing environmental pollution. However, the success of this process depends on developing suitable catalysts that can enhance both product selectivity and catalytic activity. Currently, most eNORR catalysts are metallic in nature.^13–17^ While these metal catalysts demonstrate excellent eNORR activity, their practical application is limited by factors such as poor durability, high cost, and low atom utilisation efficiency.^18,19^ Given these limitations, the search for low-cost, highly selective eNORR catalysts has become urgent and necessary.

Single-atom catalysts (SACs) have drawn remarkable attention because of their exceptional catalytic performance. Single-atoms are atomically dispersed on the surface of the substrate and existing in an unsaturated state that gives them with high activity. This unique structure provides an abundance of active sites for reactant molecules, thereby facilitating efficient catalysis.^20,21^ To date, numerous theoretical studies and experimental results have shown that the unsaturated active sites on the surface of catalyst materials, including SACs and surface defects, can effectively promote the eNORR.^22–30^ However, a notable challenge arises with (TM)-based SACs, where the high mobility of surface metal atoms often leads to easy agglomeration of single metal atoms on their surfaces.^31–33^ In contrast, surface defects are more effective and stable catalytic sites.^28–30^

Molybdenum disulfide (MoS_2_) is a typical 2D material. During the synthesis of MoS_2_ monolayer, sulfur (S) vacancies are inevitably generated on its surface, thus exposing the underlying unsaturated molybdenum (Mo) atoms.^34^ These S-vacancy sites on MoS_2_ materials can provide stable catalytic active sites for eNORR.^35^ However, in this study, we found that the intrinsic properties of monolayer MoS_2_ with S vacancies posed certain limitations. Specifically, the three unsaturated Mo atoms exhibited excessively strong binding affinity for reactant molecules, which hindered the smooth progression of some key elementary reaction steps. For instance, the energy barrier for the NH → NH_2_ step was found to be nearly 1.5 eV, indicating a significant kinetic barrier. This observation suggests that while MoS_2_ with S vacancies provides a promising foundation, further modification is necessary to optimize its catalytic performance for eNORR.

In the field of gas sensing, introducing non-metal atoms into S vacancy sites of MoS_2_ can regulate the adsorption energy of adsorbed molecules, thereby effectively improving its performance.^36,37^ Similarly, in the context of electrocatalysis, incorporating non-metal atoms into two-dimensional (2D) materials has been shown to enhance their eNORR performance. For instance, Ali *et al.*^38^ examined the eNORR performance of B_4_@g-C_3_N_4_ catalysts and discovered that B_4_ clusters anchored on g-C_3_N_4_ materials could efficiently enhance eNORR with a limiting potential of −0.37 V. Zhu *et al.*^39^ reported that a single silicon atom supported on carbon nanotubes exhibited superior catalytic activity in the eNORR, with a limiting potential of −0.25 V. Ma *et al.*^40^ investigated the eNORR performance of phosphorus (P)-doped C_2_N materials and revealed their superior catalytic activity. Meanwhile, Saeidi *et al.*^41^ demonstrated the remarkably high catalytic activity of boron-doped C_3_N nanosheets for eNORR. Taken together, these studies highlight the potential of single non-metal atoms as effective eNORR catalysts.

In view of this, the MoS_2_ monolayer with an S vacancy is an ideal support, with its S vacancy sites offering robust binding sites for anchoring non-metal heteroatoms.^42^ Leveraging this characteristic, we have constructed single non-metal atom catalysts by embedding single non-metal atoms (denoted NM) into MoS_2_ vacancies (referred to as NM@MoS_2_). It is anticipated that these NM@MoS_2_ catalysts will deliver exceptional catalytic performance in the eNORR.

It is worth noting that MoS_2_ materials doped with non-metal atoms are known for their simple and straightforward preparation process. For instance, Ma *et al.*^43^ demonstrated *via* theoretical calculations that CO, NO, and NO_2_ molecules can fill S vacancies in MoS_2_ at room temperature, thereby achieving C, N, and O doping, respectively. This finding suggests that non-metal doping can be achieved under mild conditions. Furthermore, Song *et al.*^44^ successfully synthesized phosphorus (P)-doped MoS_2_ materials *via* a simple pyrolysis process, achieving remarkable oxygen reduction reaction (ORR) performance. Xie *et al.*^45^ produced MoS_2_ materials with different oxygen doping concentrations by controlling the hydrothermal reaction temperature; these nanosheets exhibited excellent catalytic performance in the hydrogen evolution reaction (HER). Zhang *et al.*^46^ prepared selenium (Se)-doped MoS_2_ materials using a hydrothermal synthesis method. They then combined these materials with reduced graphene oxide, resulting in composites that exhibited excellent catalytic activity in lithium–sulfur (Li–S) batteries. Song *et al.*^47^ synthesized O-doped MoS_2_ catalysts *via* pyrolysis and combined them with g-C_3_N_4_, yielding materials with exceptional ORR performance. Similarly, several pieces of experimental research highlight that boron (B)-doped MoS_2_ catalysts synthesized by the hydrothermal route exhibit excellent catalytic performance in the electrocatalytic reduction of both nitrogen and nitrate.^48–50^ As the positions of S atoms were replaced by non-metal (NM) atoms during the doping process, the NM-doped MoS_2_ structure is similar to that of NM@MoS_2_ catalysts. Therefore, these research findings offer compelling theoretical support for the experimental synthesis and stability of NM@MoS_2_ catalysts.

In this study, we employed density functional theory (DFT) calculations to systematically analyse the performance of eight non-metal single-atom catalysts (NM@MoS_2_, where NM represents B, C, N, O, P, Si, Se, and Te) in the eNORR process. Firstly, we calculated the binding energies of NM@MoS_2_ catalysts to evaluate their thermodynamic stability. We then conducted a comprehensive electronic structure analysis to see how well the catalysts could adsorb and activate NO molecules. Next, we examined the eNORR performance of these catalysts in more detail, considering critical factors such as reaction pathways and product selectivity. Finally, we examined the competitive relationship between HER and eNORR. Based on this analysis, we identified potential eNORR catalysts.

## 2 Computational methods

The Vienna *Ab Initio* Simulation Package (VASP) was used for our calculations to perform density functional theory (DFT) calculations of spin polarization.^51^ The generalized gradient approximation (GGA) and Perdew–Burke–Ernzerhof (PBE) functional are used to describe the electron exchange and correlation terms.^52^ The Projector Augmented Wave (PAW) was used to describe the ion–electron interactions with the energy cutoff set to 450 eV.^53^ All atoms were fully relaxed, and the standard parameters for convergence of the energies and forces were set to 10^−4^ eV and 0.02 eV Å^−1^. The DFT-D3 method was used to correct for the weak interactions between adsorbates and surfaces (van der Waals interactions).^54^ A 4 × 4 × 1 Monkhorst–Pack *k*-point grid was used to sample the Brillouin zone during geometry optimization.^55^ A 4 × 4 × 1 single-layer supercell is constructed to model the catalyst surface. In order to eliminate the potential influence of periodic boundary conditions on the calculation results, a vacuum layer with a thickness of 15 Å was set in the *Z* direction of the lattice. To test the feasibility of doping MoS_2_ with non-metal atoms (NM), the binding energy (*E*_bind_) was calculated according to eqn (1):

1 *E*_b_ = *E*_NM@MoS_2__ − *E*_defect_ − *µ*_NM_

Here *E*_NM@MoS_2__ and *E*_defective-MoS_2__ represent the total energies of NM atoms deposited on MoS_2_ and MoS_2_ with one sulphur vacancy, respectively. *µ*_NM_ denotes the chemical potential of the NM atoms. For N and O atoms, it is obtained from their gas-phase (N_2_ and O_2_) energies per atom. The chemical potential of all other atoms are obtained from their bulk or solid phase (β-rhombohedral boron for B, graphite for C, black phosphorus for P, diamond-cubic silicon for Si, grey selenium for Se, and hexagonal tellurium for Te, respectively) energies per atom. In view of the fact that the reaction process involves the transfer of protons and electrons (NO + 5H^+^ + 5e^−^ → NH_3_ + H_2_O), we calculate the Gibbs free energy (Δ*G*) by means of the computational hydrogen electrode (CHE) model, where the free energy of the H^+^ + e^−^ is considered to be half of the chemical potential of the hydrogen gas under standard conditions.^56^ The standard conditions for the reaction are *P*_H_2__ = 1 bar and a temperature of 298.15 K. Δ*G* of the eNORR step was calculated using eqn (2):

2 Δ*G* = Δ*E* + Δ*G*_*U*_ + Δ*G*_pH_ + Δ*E*_ZPE_ − *T*Δ*S*

In this equation, Δ*E* represents the energy change between reactants and products, which can be calculated directly from DFT. Δ*E*_ZPE_ and *T*Δ*S* are the zero-point energy and entropy changes at room temperature (*T* = 298.15 K), respectively, which can be calculated from vibrational frequencies *via* the VASPKIT package.^57^ Δ*G*_pH_ is the pH-induced free energy change, calculated as Δ*G*_pH_ = *K*_B_*T* × pH × ln 10, where *K*_B_ is the Boltzmann constant and pH is set to zero. Δ*G*_*U*_ is the correction of Δ*G* by the electrode potential *U*, Δ*G*_*U*_ = −*eU*, *e* is the number of electrons transferred at each step and *U* is the applied voltage.

The limiting potential (*U*_L_) is a measure of catalytic activity, calculated using eqn (3) to determine the minimum voltage required to make each reaction step exothermic.^36,37^

3 *U*_L_ = −(Δ*G*_1_, Δ*G*_2_, Δ*G*_3_, …, Δ*G*_*i*_)_max_/*e*

Here, Δ*G*_*i*_ denotes the Gibbs free energy for each step of the radical reaction in the eNORR process. According to this definition, the more positive the *U*_L_ of the catalyst, the smaller the applied voltage, indicating the higher catalytic activity. In addition, the effect caused by the solvent on Δ*G* was simulated by the Poisson–Boltzmann (PB) implicit solvent model in the VASP software package.^58^

## 3 Results and discussion

### 3.1. NM@MoS_2_ stability

**Fig. 1:**
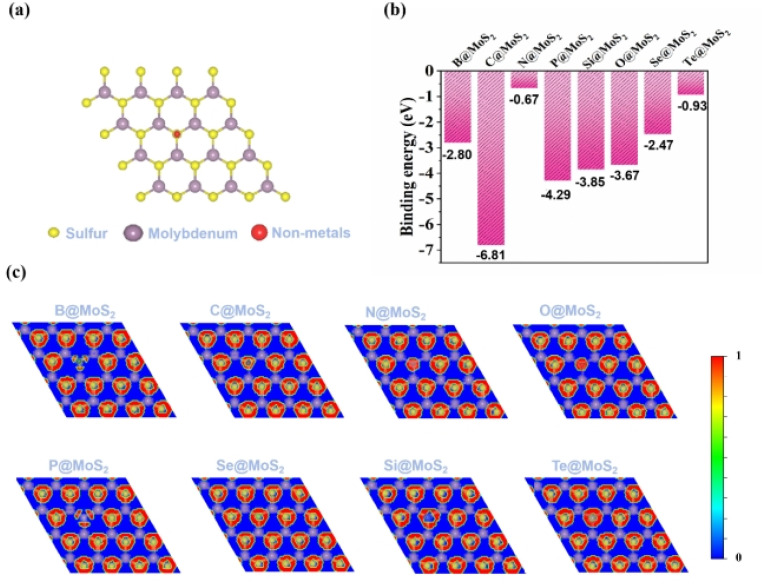
(a) The modeled structure of NM@MoS_2_ catalysts (top); (b) binding energies of NM@MoS_2_ catalysts; (c) ELF diagram of NM@MoS_2_ catalyst.

For our study, we precisely substituted all eight non-metallic (NM) atoms, namely B, C, N, O, P, Si, Se, and Te, into sulfur (S) vacancy defects in the MoS_2_ substrate, as illustrated in Fig. 1a. To quantitatively evaluate the stability and experimental feasibility of the eight NM@MoS_2_ catalysts, we first calculated their binding energies. By definition, a more negative *E*_bind_ indicates a stronger and more stable interaction between the NM atoms and MoS_2_ substrate. As depicted in Fig. 1b, all NM@MoS_2_ catalysts showed negative *E*_bind_ values ranging from −0.67 to −6.81 eV, thereby confirming their stability. In addition, the stability of the catalysts was further corroborated by calculation of the electronic localization function (ELF),^59^ which elucidates the bonding characteristics between the NM atoms and MoS_2_. The ELF value of 1 (represented by the red region) indicates the presence of a strong covalent bond. As shown in Fig. 1c, there is a significant overlap of electron density between the NM atoms and the Mo atoms, with the NM–Mo bond situated in the red region, indicating the presence of covalent bond components between NM and Mo.

**Fig. 2:**
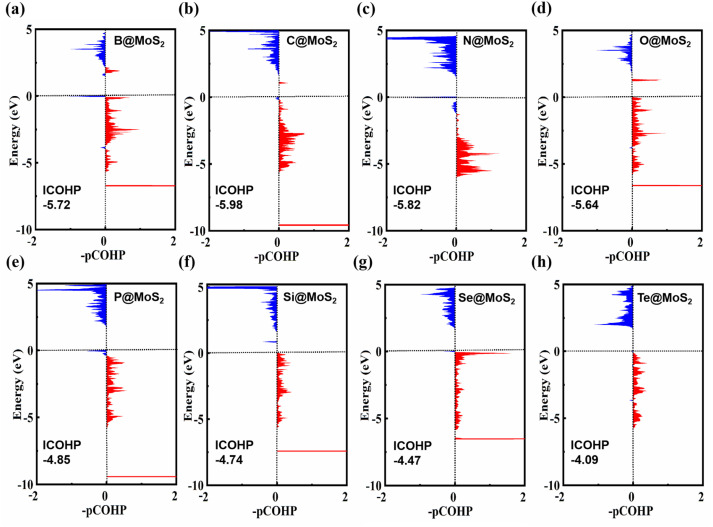
COHP describes the interaction between the NM and Mo in the NM–Mo bond, while ICOHP represents the strength of this bond (the dashed line corresponds to the Fermi level). (a) B@MoS_2_; (b) C@MoS_2_; (c) N@MoS_2_; (d) O@MoS_2_; (e) P@MoS_2_; (f) Si@MoS_2_; (g) Se@MoS_2_; (h) Te@MoS_2_.

We further elucidate the bonding characteristics between NM and Mo by calculating the projected (pCOHP) and integral (ICOHP) crystal orbital Hamilton populations (see Fig. 2).^60^ The left side corresponds to the antibonding orbitals of NM–Mo and the right side to the bonding orbitals in pCOHP. Notably, below the Fermi level, the NM–Mo bond states are predominantly found in the low-energy bonding region, suggesting the stability of the NM–Mo bond. Furthermore, a quantitative assessment of the NM–Mo bond strength using the ICOHP method revealed that, with the exception of the Se@MoS_2_ (−4.47) and Te@MoS_2_ (−4.09) systems, the ICOHP values for the B@MoS_2_ (−5.72), C@MoS_2_ (−5.98), N@MoS_2_ (−5.82), O@MoS_2_ (−5.64), P@MoS_2_ (−4.85) and Si@MoS_2_ (−4.74) systems were all lower than that of pristine MoS_2_ (−4.70). This implies that, in addition to the systems doped with Se and Te, NM–Mo bonds are stronger than S–Mo bonds. This demonstrates that covalent bonds are also formed between the NM (B, C, N, O, P and Si) atoms and the surrounding Mo atoms. Consequently, NM@MoS_2_ can serve as a stable electrocatalyst in the eNORR process.

### 3.2. NO adsorption and activation

**Fig. 3:**
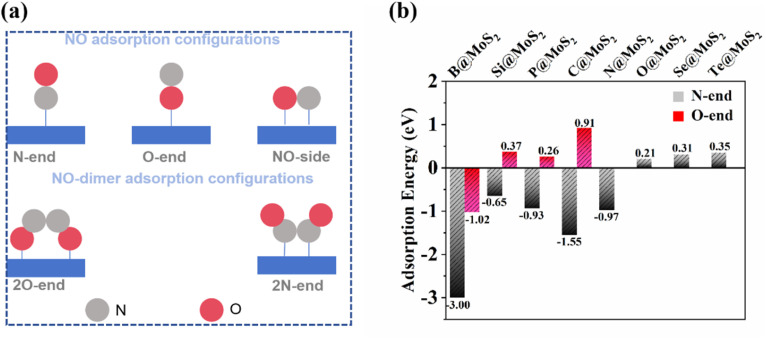
(a) Possible NO adsoprtion configurations both high and low NO concentration; (b) NO adsorption energy on NM@MoS_2_ catalyst.

The adsorption and activation of NO molecules are pivotal steps in initiating the eNORR and significantly influence the subsequent protonation process. In this study, we thoroughly investigated three potential adsorption configurations of NO molecules on the surface of NM@MoS_2_ catalysts: N-end, O-end, and NO-side, as illustrated in Fig. 3a. It is important to highlight that for the O@MoS_2_, Se@MoS_2_, and Te@MoS_2_ catalysts, the N-end configurations stable, yet they only exhibit physical adsorption of NO molecules, as evidenced by their Δ*G* (*NO) values being greater than zero (Fig. 3b). Given their insufficient adsorption strength for NO molecules, these catalysts were deemed unsuitable for the eNORR process and were thus excluded from the list of potential eNORR candidates.

For the remaining five NM@MoS_2_ catalysts, NO molecules preferentially adsorb in the N-end configurations on the catalyst surface, exhibiting the smallest Δ*G* (*NO) values. Notably, after structural optimization, the NO-side adsorption configurations become unstable and spontaneously transition to the N-end configurations. Once adsorbed in the N-end configuration, the N=O bond lengths increase to 1.19 Å (B@MoS_2_), 1.20 Å (C@MoS_2_), 1.20 Å (N@MoS_2_), 1.20 Å (Si@MoS_2_), and 1.23 Å (P@MoS_2_), respectively. These values are significantly longer than the bond length of 1.16 Å in the free NO molecule, indicating that the NO molecule is effectively activated upon adsorption onto the catalyst surface.

To further elucidate the active origins of these catalysts, we conducted detailed electronic structure calculations, including charge density difference (CDD) and Bader charge analysis,^61,62^ to reveal the charge distribution between the active sites and adsorbed NO (*NO) molecules. These analyses help explore the underlying interaction mechanisms. Additionally, we employed pCOHP, ICOHP, and partial density of states (PDOS) to gain deeper insights into the orbital contributions and chemical bonding characteristics at the active sites. This systematic study provides a comprehensive theoretical foundation for understanding the fundamental origins of the catalysts activity.

**Fig. 4:**
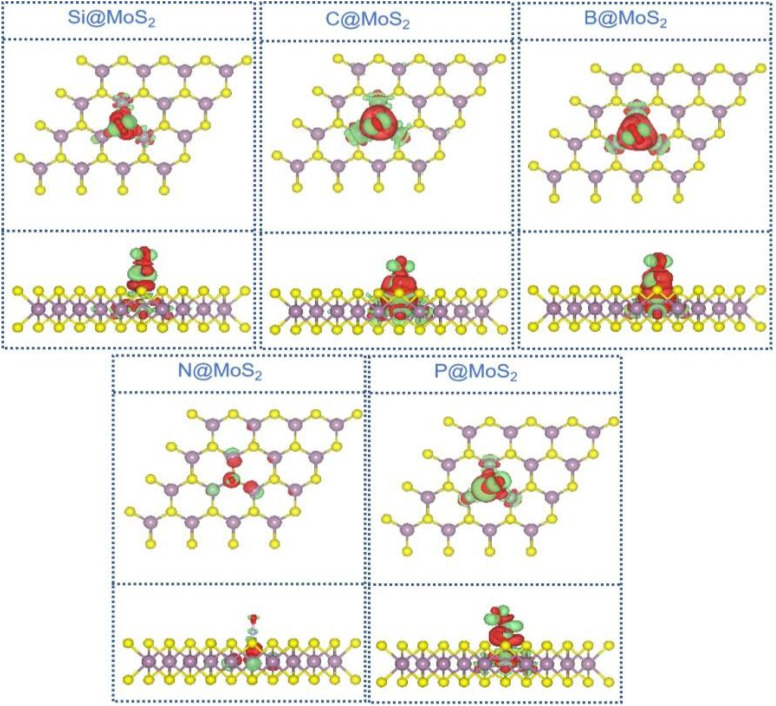
CDD diagram of NO adsorption on NM@MoS_2_ catalyst. The isosurface value is set to 0.002 e Å^−3^.

As depicted in Fig. 4, we plotted the CDD diagram for NO adsorption on NM@MoS_2_ catalysts. The green areas signify electron accumulation, while the red areas indicate electron depletion. A significant charge redistribution occurs between the *NO and the catalyst surface. Specifically, electrons are primarily concentrated on the *NO molecule, while the neighboring non-metal (NM) atoms lose electrons. Bader charge analysis further confirms this phenomenon, revealing that electrons are transferred from the NM@MoS_2_ catalyst to the *NO molecule, with the transferred electron amounts being 0.75*e* (B@MoS_2_), 0.73*e* (Si@MoS_2_), 0.70*e* (P@MoS_2_), 0.62*e* (C@MoS_2_), and 0.04*e* (N@MoS_2_). Additionally, a substantial electron density is concentrated between the NM atoms and the *NO molecule, suggesting that the newly formed NM–N bond exhibits covalent bonding characteristics.

**Fig. 5:**
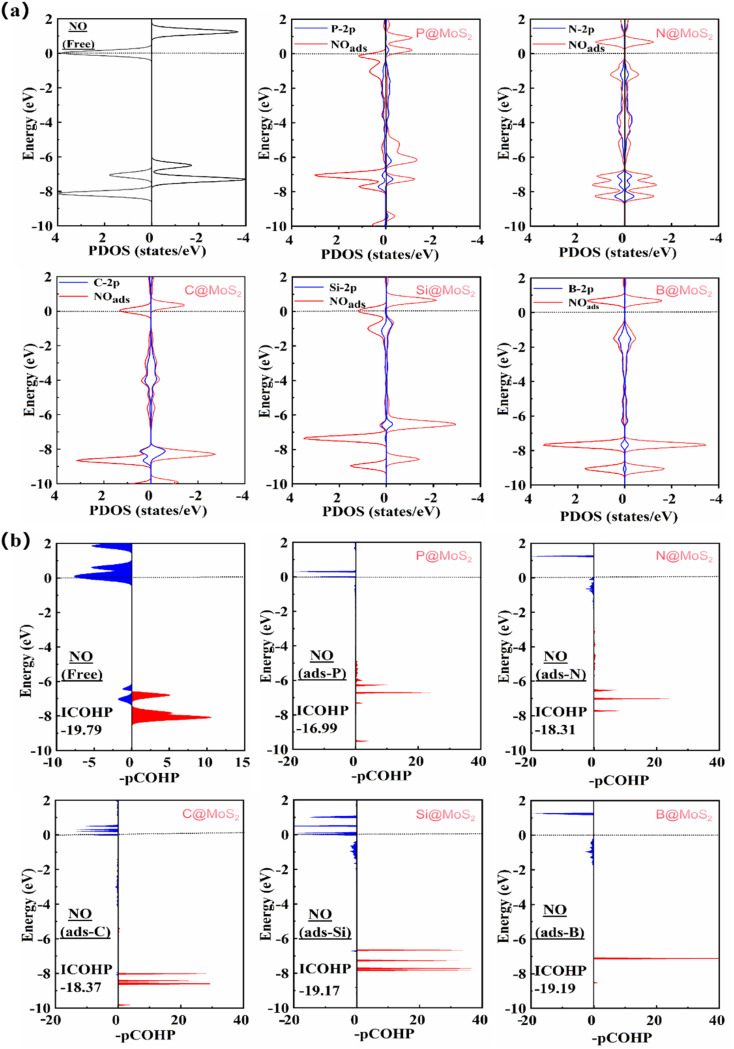
(a) PDOS diagram of NO adsorption on NM@MoS_2_ catalyst; (b) the COHP diagram of NO before and after adsorption. The left side corresponds to the antibonding orbital of the N=O bond and the right side to the bonding orbital. The labelled value of ICOHP indicates the strength of the N–O bond in the N=O bond.

Fig. 5a presents the PDOS plots for NO molecules after adsorption on different catalysts. It is evident that there is significant orbital mixing between the NM-2p orbitals and the NO-2π* orbitals near the Fermi energy level, indicating a strong interaction between the NO molecules and the NM atoms. Moreover, the energy of the NO-2π* orbitals decreases upon adsorption due to electrons transfer from the NM-2p orbitals.

To further investigate NO activation, we plotted the COHP diagram for NO adsorption on NM@MoS_2_ catalysts, as shown in Fig. 5b. Upon adsorption of NO molecules on NM@MoS_2_ catalysts, the antibonding orbitals of the N=O bonds shift downward near the Fermi energy level, resulting in more antibonding states of the N=O bonds below the Fermi energy level. The integral COHP (ICOHP) values, which quantify the strength of chemical bonds, are −16.99 (P@MoS_2_), −18.31 (N@MoS_2_), −18.37 (C@MoS_2_), −19.17 (Si@MoS_2_), and −19.19 (B@MoS_2_). These values are less negative compared to −19.79 for the free NO molecule, indicating effective activation of the N=O bonds upon NO adsorption.

Based on these findings, non-metal atoms act as active sites, effectively adsorbing and activating NO molecules. Given that the N-end adsorption configurations of NO molecules is more stable, we primarily focus on discussing the eNORR behavior of NO in the N-end adsorption configuration.

### 3.3. eNORR mechanism and activity

**Fig. 6:**
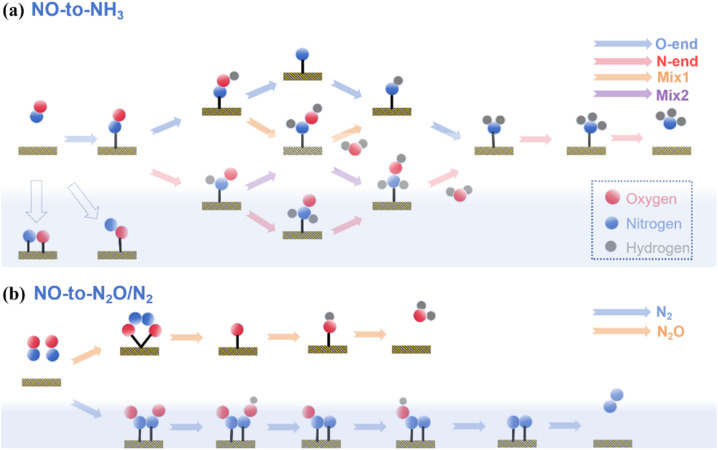
Schematic illustration of possible eNORR reaction pathways; (a) pathway to the formation of NH_3_; (b) pathway to the formation of N_2_O or N_2_.

The eNORR reaction can proceed through several potential reduction pathways, and the coverage of NO molecules plays a crucial role in determining product selectivity. As shown in Fig. 6a, at low NO coverage, the reaction involves five proton-coupled electron transfer steps, ultimately yielding NH_3_. By contrast, at high NO coverage, the reaction is driven by the dimerisation of NO molecules to form N_2_O_2_, resulting in the production of N_2_O or N_2_ as by-products.^63^

**Fig. 7:**
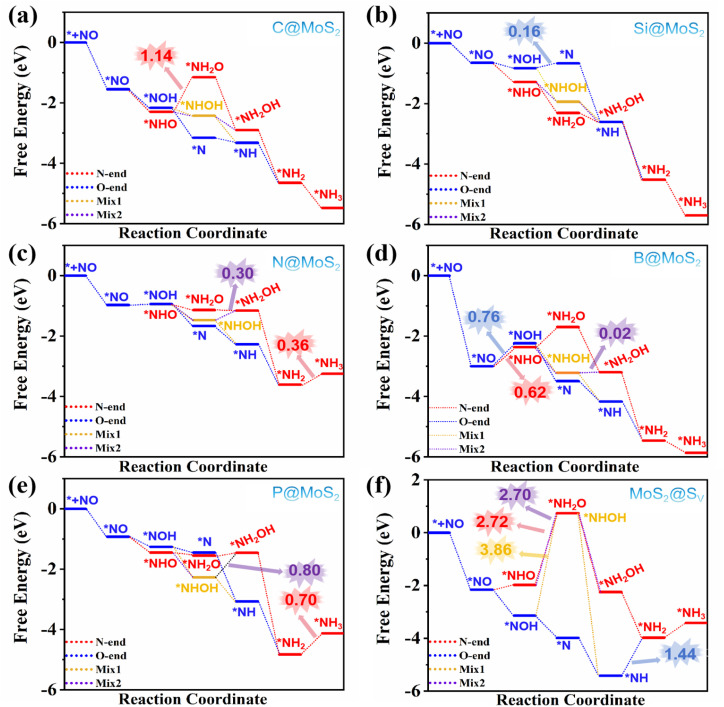
Free energy diagrams of eNORR to NH_3_ over five NM@MoS_2_ catalysts and one MoS_2_@V_S_ catalysts; (a) C@MoS_2_; (b) Si@MoS_2_; (c) N@MoS_2_; (d) B@MoS_2_; (e) P@MoS_2_; (f) MoS_2_@S_v_.

We evaluated the catalytic performance of B@MoS_2_, C@MoS_2_, P@MoS_2_, Si@MoS_2_, and N@MoS_2_ catalysts in the eNORR for NH_3_ synthesis based on the possible pathways shown in the reaction schematic in Fig. 6a. The results are summarized in Table S1 and Fig. 7. As shown in Fig. 7, the eNORR process begins with the adsorption of NO molecules to form *NO species. Subsequently, the protons in the electrolyte react with *NO to form *HNO or *NOH intermediates. Then sequential hydrogenation processes occur, finally yielding NH_3_ and H_2_O.

As shown in Fig. 7a, the most selective pathway for the C@MoS_2_ catalyst is Mix2, with all elementary steps being exergonic (Δ*G* < 0 eV). This suggests that the eNORR process can proceed spontaneously. As a result, the limiting potential (*U*_L_) for the C@MoS_2_ catalyst is 0 V. For the Si@MoS_2_ catalyst, the most favorable path is the N-end path depicted in Fig. 7b. In this pathway, the energy of each intermediate species decreases, enabling the eNORR process to occur at 0 V, meaning that the *U*_L_ for the Si@MoS_2_ catalyst is also 0 V. For the N@MoS_2_ catalyst, the optimal reaction path is the N-end path (Fig. 7c). Here, the energies of all elementary steps decrease, except for the final step (*NH_2_ → *NH_3_), which exhibits an energy increase (Δ*G* = 0.36 eV). This step is identified as the RDS, with a corresponding *U*_L_ of −0.36 V. For the B@MoS_2_ catalyst (Fig. 7d), the optimal reaction pathway is Mix1. Along this pathway, overcoming the energy barrier of 0.62 eV (Δ*G* = 0.62 eV) in the *NO → *NHO step is the RDS, resulting in a *U*_L_ of −0.62 V. Similarly, for the P@MoS_2_ catalyst, the Mix1 pathway is identified as the optimal reaction pathway (Fig. 7e). Here, the energy of all elementary steps decreases, except for the NH_2_ → NH_3_ step, which has an energy barrier of 0.70 eV. This step is the RDS, with a *U*_L_ of −0.70 V. Under acidic conditions, the conversion of NH_3_ to NH_4_^+^ is typically exergonic.^64^ Therefore, the catalytic activity of the NM@MoS_2_ catalysts for eNORR to NH_3_ was evaluated without considering NH_3_ desorption further.

Since no thermodynamic energy barrier was observed on the C@MoS_2_ and Si@MoS_2_ catalysts. Taking the C@MoS_2_ catalyst as an example, we employed the climbing-image nudged elastic band (CI-NEB) method to calculate the energy barriers for each elementary step along the optimal reaction path of Mix2. The energy barriers (*E*_B_) for all elementary reaction steps are shown in Fig. S1. As shown in Fig. S1, the energy barrier (0.92 eV) for the third hydrogenation step (NHOH + H^+^ + e^−^ → *NH_2_OH) is higher than for the other steps. Consequently, this process constitutes the kinetically rate-determining step of this reaction pathway. However, from NO(g) to the NH_2_OH species, all elementary reaction steps are exergonic, with a total energy of −2.89 eV. This value is higher than the kinetic energy barrier (0.92 eV), suggesting that the energies released from the previous elementary reactions are sufficient to overcome the energy barrier.

To further elucidate the impact of NM atom modification, we examined the eNORR activity of MoS_2_ containing sulfur vacancies (MoS_2_@V_S_). For the MoS_2_@V_S_ catalyst, the most favorable path is the O-end pathway, as depicted in Fig. 7f. Along this pathway, the *NH → *NH_2_ process is the RDS with a *U*_L_ value of −1.44 V. This demonstrates that the *U*_L_ values of the NM@MoS_2_ catalysts are substantially altered compared to the unmodified MoS_2_@V_S_ catalyst: the *U*_L_ values of C@MoS_2_ and Si@MoS_2_ are both elevated to 0 V; the *U*_L_ value of N@MoS_2_ is increased to −0.36 V; the *U*_L_ value of B@MoS_2_ is elevated to −0.62 V; and the *U*_L_ value of P@MoS_2_ is increased to −0.70 V. These analyses clearly demonstrate that NM atom modification dramatically enhances the eNORR activity of MoS_2_ catalysts.

Based on the above analysis, the catalytic performance of the C@MoS_2_ and Si@MoS_2_ catalysts is superior to that of the B@MoS_2_, N@MoS_2_, and P@MoS_2_ catalysts. To elucidate the differences in catalytic activity, we conducted an electronic structure analysis of each catalyst. Fig. S2 shows the band structures of N@MoS_2_, P@MoS_2_, B@MoS_2_, C@MoS_2_, and Si@MoS_2_. Notably, the bandgap widths of C@MoS_2_ (0.97 eV) and Si@MoS_2_ (0.78 eV) are significantly smaller than those of N@MoS_2_ (1.62 eV), P@MoS_2_ (1.54 eV), and B@MoS_2_ (1.37 eV). Consequently, C@MoS_2_ and Si@MoS_2_ exhibit higher electron mobility throughout the eNORR process. This optimizes the adsorption of reaction intermediates and reduces the energy barriers of key steps, such as*NO → *NHO or *NH_2_ → *NH_3_, thereby significantly enhancing eNORR activity.

It has been well-documented that at high NO concentrations, *NO molecules can form dimers (*NONO), as illustrated in the configuration shown in Fig. 3a. These *NONO dimers can adsorb onto the catalyst surface in either a 2O-side or 2N-side configuration, leading to the formation of by-products such as N_2_O or N_2_ (Fig. 6b shows possible reaction pathways).^63^ To further investigate the catalytic behavior of eNORR at high NO concentrations, we conducted a detailed analysis of the catalytic activity for this reaction (see Table S2).

For the C@MoS_2_, P@MoS_2_, and N@MoS_2_ catalysts, the high electronegativity of the C, P and N elements significantly inhibits electron transfer during adsorption. This results in an unstable 2N-side adsorption configuration of NONO, thereby preventing the side reaction that leads to N_2_ formation. Additionally, the N@MoS_2_ catalyst lacks an O-end adsorption configuration for NO monomers, which prevents *NONO from adsorbing in the 2O-side configuration.^65^ Consequently, the N@MoS_2_ catalyst exclusively produces NH_3_ as the main product while effectively suppressing the formation of N_2_O and N_2_ by-products.

**Fig. 8:**
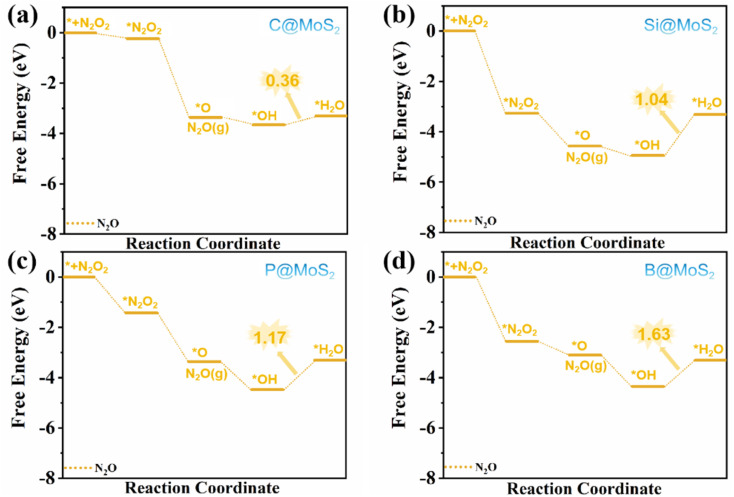
Free energy diagrams of eNORR to N_2_O over four NM@MoS_2_ catalysts; (a) C@MoS_2_; (b) Si@MoS_2_; (c) P@MoS_2_; (d) B@MoS_2_.

In contrast, on B@MoS_2_ and Si@MoS_2_ catalysts, we discovered that *NONO has a preference for adsorbing on the 2O-side configuration by contrasting the adsorption energies of *NONO in two different configurations. This selective adsorption configuration inhibits the formation of N_2_, thereby reducing the likelihood of N_2_ being produced as a by-product. Therefore, we focused on the reaction pathway from *NONO to N_2_O over the C@MoS_2_, P@MoS_2_, B@MoS_2_, and Si@MoS_2_ catalysts, as shown in Fig. 8.

As depicted in Fig. 8, the rate-determining step (RDS) for N_2_O formation is the conversion of OH to H_2_O. The corresponding limiting potentials (*U*_L_) for this process are −0.36 V (C@MoS_2_), −1.04 V (Si@MoS_2_), −1.17 V (P@MoS_2_), and −1.63 V (B@MoS_2_). Based on these results, the aforementioned catalysts exhibit high selectivity for NH_3_, even at high NO coverage, as evidenced by *U*_L_ (NH_3_) > *U*_L_ (N_2_O). Furthermore, the N@MoS_2_ catalyst demonstrates high selectivity for NH_3_ given that the formation of *NONO is unfavourable on this catalyst.

In summary, the C@MoS_2_, P@MoS_2_, B@MoS_2_, Si@MoS_2_, and N@MoS_2_ catalysts all demonstrate excellent selectivity for NH_3_ products under conditions of high NO coverage. This suggests that these catalysts are highly promising for the efficient synthesis of NH_3_*via* the eNORR process.

### 3.4. eNORR with HER selectivity

**Fig. 9:**
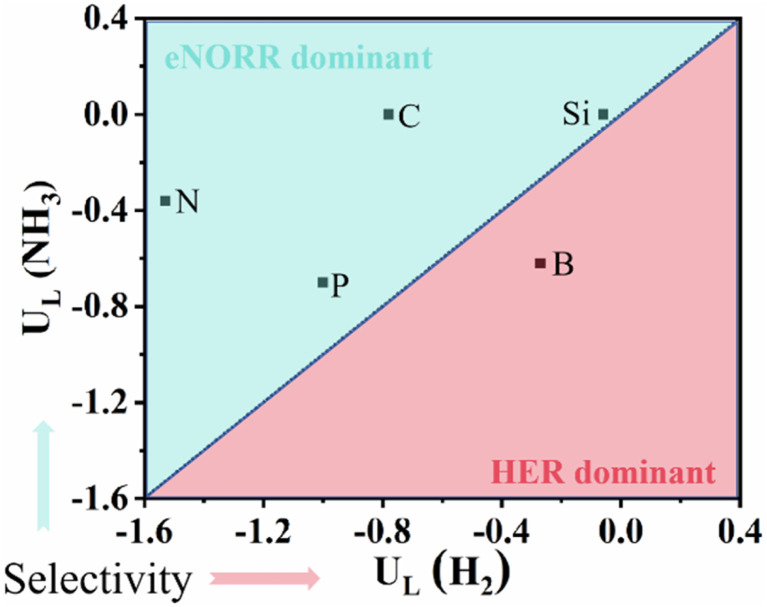
The *U*_L_(NH_3_) *versus* the *U*_L_(HER).

In aqueous solutions, the HER is the main competitive process for the eNORR.^66^ Thus, we compare the *U*_L_ (NH_3_) and *U*_L_ (H_2_) values for the five NM@MoS_2_ catalysts (see Fig. 9).

As shown in Fig. 9, the *U*_L_ (H_2_) values on the five catalysts are as follows: −0.06 V (Si@MoS_2_), −0.27 V (B@MoS_2_), −0.78 V (C@MoS_2_), −1.00 V (P@MoS_2_), and −1.53 V (N@MoS_2_). Our calculations reveal that, for P@MoS_2_, N@MoS_2_, C@MoS_2_, and Si@MoS_2_, the *U*_L_ (NH_3_) values are notably higher than the *U*_L_ (H_2_) values. At the initial potential, eNORR can effectively inhibit the occurrence of HER, thereby enhancing the selectivity and efficiency of eNORR.

Consequently, N, P, C, and Si@MoS_2_ catalysts demonstrate superior performance in prioritising eNORR over HER, rendering them highly promising candidates for efficient NH_3_ synthesis *via* eNORR.

## 4 Conclusions

In summary, we used DFT calculations to identify and screen potential catalysts for NH_3_ synthesis *via* the eNORR. We focused on eight thermodynamically stable NM@MoS_2_ catalysts (where NM = B, C, N, O, P, Si, Se, or Te), evaluating their ability to adsorb and activate NO molecules. Our computational results revealed that five of the catalysts (those with NM = B, C, N, P and Si) exhibited effective adsorption and activation of NO molecules and significant potential for the eNORR, with *U*_L_ values of 0 V for Si@MoS_2_ and C@MoS_2_, −0.36 V for N@MoS_2_, −0.62 V for B@MoS_2_, and −0.70 V for P@MoS_2_. These catalysts also exhibited high NH_3_ selectivity by suppressing HER competition and minimizing side reactions. These results demonstrate that NM@MoS_2_ catalysts excel in terms of stability, selectivity and catalytic activity, making them highly promising eNORR candidates. This study offers fresh perspectives on the management of NO and the synthesis of NH_3_, and could pave the way for more efficient and sustainable catalytic processes.

## Author contributions

Yifan Liu: data curation, formal analysis, writing – original draft. Mamutjan Tursun: conceptualization, data curation, writing – review & editing. Guangzhi Hu: writing – review & editing. Abdukader Abdukayum: writing – review & editing. Chao Wu: software, computing resource, writing – review & editing.

## Conflicts of interest

There are no conflicts to declare.

## Supplementary Material

RA-015-D5RA04718H-s001

## Data Availability

The data supporting this article have been included as part of the SI. Supplementary information is available: Energy Barriers (*E*_B_) of each elementary reaction steps for C@MoS_2_; band structures of NM@MoS_2 _catalysts; at low NO coverage, free energy changes (Δ*G*) of all eNORR elementary steps for NM@MoS_2_ (NM = B, C, N, P, and Si); at high NO coverage, free energy changes (Δ*G*) of all eNORR elementary steps for NM@MoS_2_ (NM = B, C, N, P, and Si). See DOI: https://doi.org/10.1039/d5ra04718h.
