# Supplementary material for: Non-metal single atoms anchored on defective MoS2: a novel electrocatalyst for NO reduction to NH3
Source: RSC Adv. 2025 Aug 19;15(36):29323–34. doi: 10.1039/d5ra04718h (PMC12377346; doi:10.1039/d5ra04718h)
Supplement: RA-015-D5RA04718H-s001 [file RA-015-D5RA04718H-s001.pdf]

Supplementary Information (SI) for RSC Advances.
This journal is © The Royal Society of Chemistry 2025

Supplementary Material

Non-Metal Single Atoms Anchored on Defective MoS_2: A Novel Electrocatalyst for NO Reduction to NH_3

Yifan Liu [a], Mamutjan Tursun [a*], Guangzhi Hu [a,b], Abdukader Abdukayum [a*], Chao Wu [c*]

[a] Xinjiang Key Laboratory of Novel Functional Materials Chemistry, College of chemistry and Environmental Sciences, Kashi University, Kashi 844000, PR China.

[b] Qilu Lake Field Scientific Observation and Research Station for Plateau Shallow Lake in Yunnan Province, Institute for Ecological Research and Pollution Control of Plateau Lakes, School of Ecology and Environmental Science, Yunnan University, Kunming 650504, China

[c] Frontier Institute of Science and Technology, Xi'an Jiaotong University, Xi'an 710054, PR China.

Contents

Figure S1 Energy Barriers (EB) of each elementary reaction steps for C@MoS_2 ......S1

Figure S2 Band structures of NM@MoS_2 catalysts. S2

Table S1 At low NO coverage, free energy changes (ΔG) of all eNORR elementary steps for NM@MoS_2 (NM=B, C, N, P, and Si)..S3

Table S2 At high NO coverage, free energy changes (ΔG) of all eNORR elementary steps for NM@MoS_2 (NM=B, C, N, P, and Si)..S4

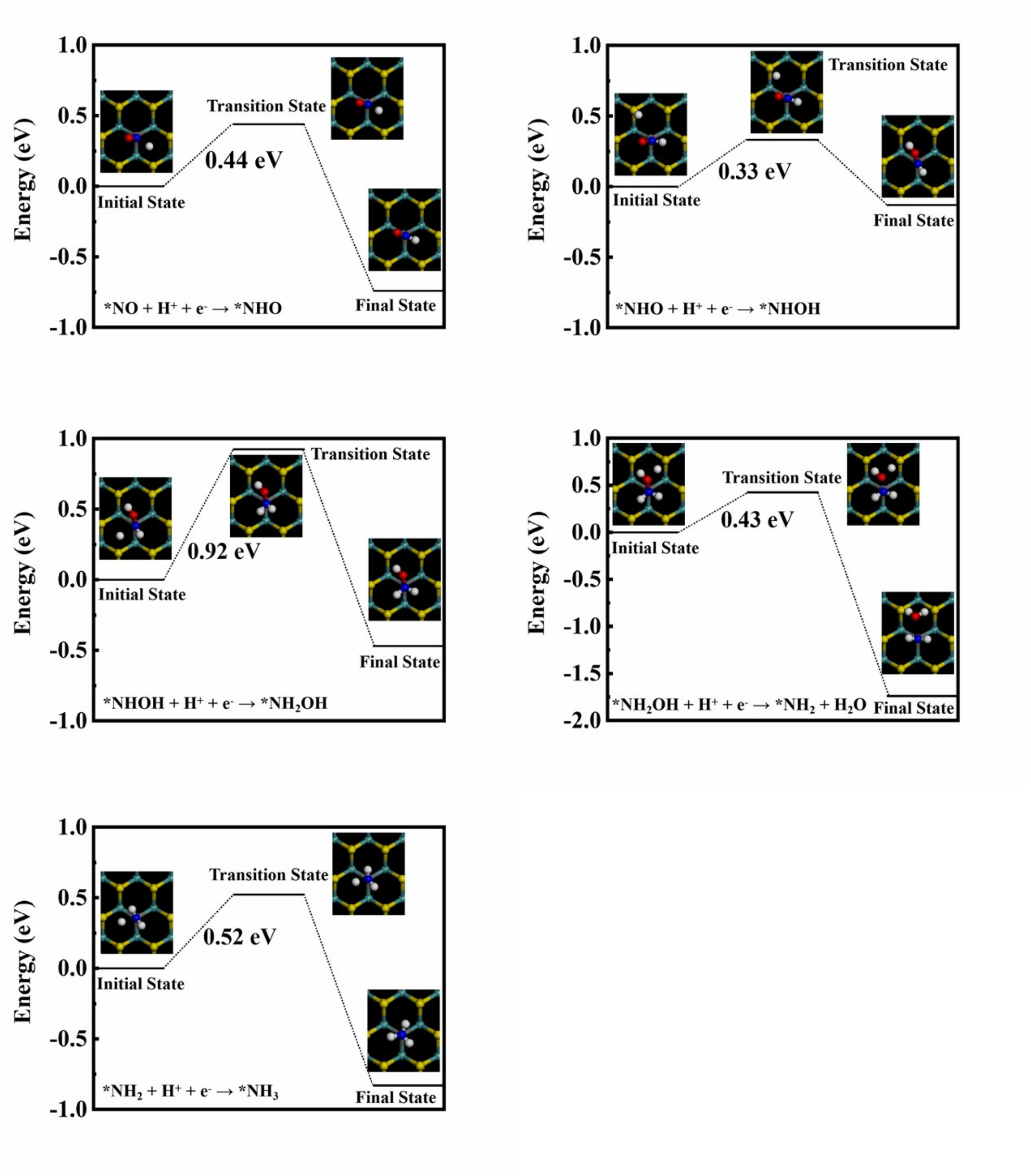

Figure S1 Energy Barriers (E_B) of each elementary reaction steps for C@MoS_2.

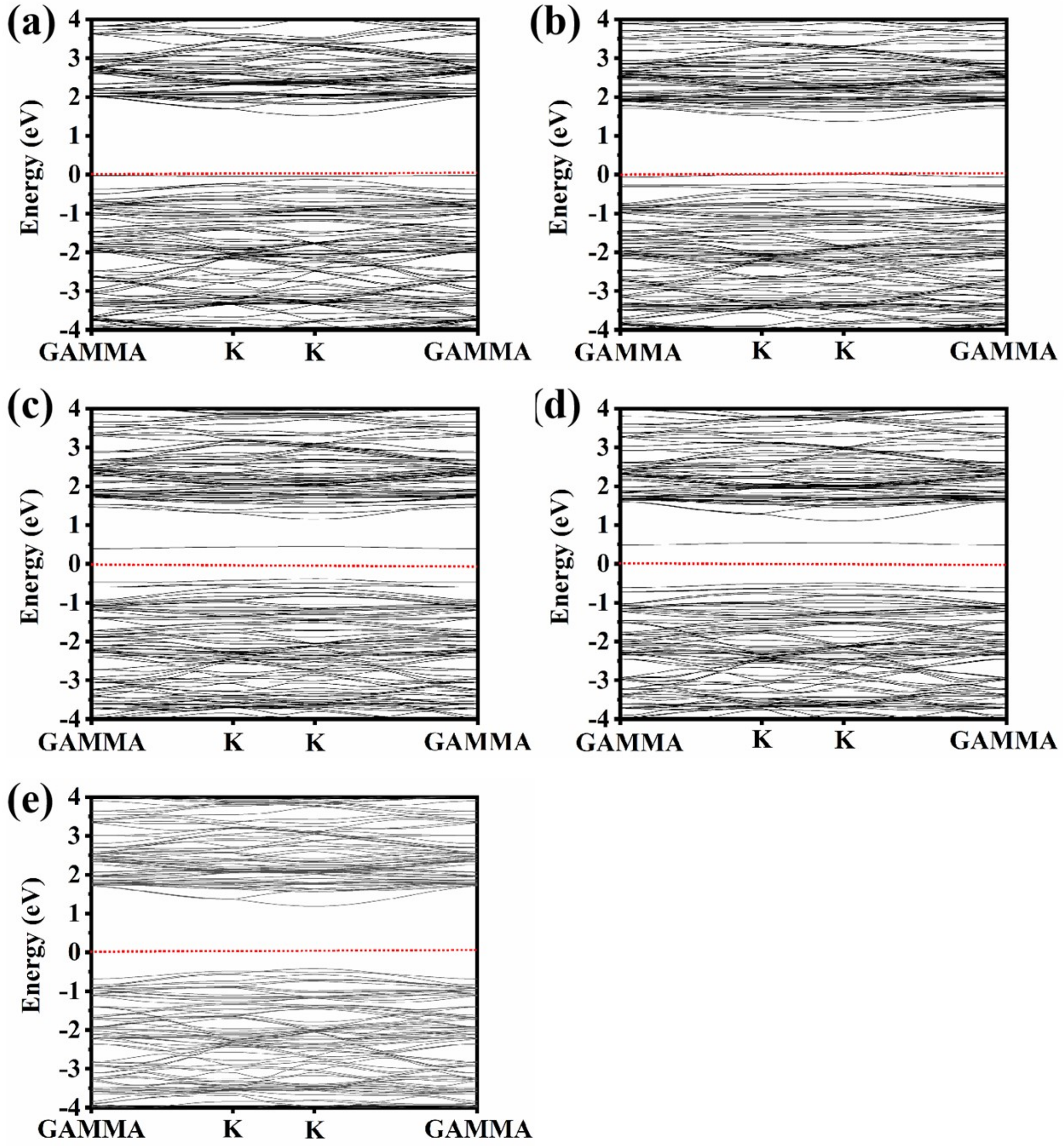

Figure S2 Band structures of (a) P@MoS_2, (b) B@MoS_2, (c) Si@MoS_2, (d) C@MoS_2, and (e) N@MoS_2 catalysts. (The red dotted line represents the Fermi level.)

Table S1 At low NO coverage, free energy changes (ΔG) of all eNORR elementary steps for NM@MoS_2 (NM=B, C, N, P, and Si).

ΔG (eV)	B	C	N	P	Si
*+NO→*NO (N-end)	-3.00	-1.55	-0.97	-0.93	-0.65
*NO+H^++e^-→*NOH	0.75	-0.61	0.03	-0.32	-0.17
*NO+H^++e^-→*NHO	0.62	-0.74	-0.02	-0.51	-0.63
*NOH+H^++e^-→*N+H_2O	-1.25	-0.99	-0.73	-0.18	0.15
*NOH+H^++e^-→*NHOH	-0.98	-0.26	-0.52	-1.00	-1.11
*NHO+H^++e^-→*NHOH	-0.85	-0.13	-0.47	-0.81	-0.65
NHO+H^++e^-→NH_2O	0.67	1.14	-0.14	-0.09	-1.01
*N+H^++e^-→*NH	-0.67	-0.15	-0.59	-1.62	-1.94
*NHOH+H^++e^-→*NH+H_2O	-0.94	-0.88	-0.79	-0.80	-0.67
NHOH+H^++e^-→NH_2OH	0.02	-0.47	0.30	0.80	-0.70
NH_2OH+H^++e^-→NH_2+H_2O	-2.26	-1.74	-2.44	-3.36	-1.87
NH+H^++e^-→NH_2	-1.29	-1.33	-1.34	-1.75	-1.90
NH_2O+H^++e^-→NH_2OH	-1.50	-1.74	-0.02	0.08	-0.33
NH_2+H^++e^-→NH_3	-0.41	-0.83	0.36	0.70	-1.18
NH_3→+NH_3	2.25	1.86	-0.36	0.51	2.07

Table S2 At high NO coverage, free energy changes (ΔG) of all eNORR elementary steps for NM@MoS_2 (NM=B, C, N, P, and Si).

ΔG (eV)	B	C	P	Si	N
$*+2NO \rightarrow *N_2O_2$(2N-end)	-1.28	/	/	-0.83	/
$*N_2O_2+H^++e^- \rightarrow *NONOH$	-1.53	//	//	-1.16	//
$*NONOH+H^++e^- \rightarrow *N_2O$	-1.72	//	//	-1.66	//
$*N_2O+H^++e^- \rightarrow *N_2OH$	-0.73	//	//	-0.27	//
$*N_2OH+H^++e^- \rightarrow *N_2$	-1.88	//	//	-2.12	//
$*+2NO \rightarrow *N_2O_2$(2O-end)	-3.27	-0.24	-1.43	-2.56	/
$*N_2O_2+H^++e^- \rightarrow *O$	-1.30	-3.13	-1.93	-0.53	//
$*O+H^++e^- \rightarrow *OH$	-0.36	-0.29	-1.10	-1.25	//
$*OH+H^++e^- \rightarrow *H_2O$	1.62	0.35	1.16	1.04	//

The notation "/" indicates that the configuration is unstable and therefore not considered, whereas "//" indicates that subsequent hydrogenation steps are not taken into account.
